# Assessment of Vascular Dysfunction in Patients Without Obstructive Coronary Artery Disease: Why, How, and When

**DOI:** 10.1016/j.jcin.2020.05.052

**Published:** 2020-08-24

**Authors:** Thomas J. Ford, Peter Ong, Udo Sechtem, John Beltrame, Paolo G. Camici, Filippo Crea, Juan-Carlos Kaski, C. Noel Bairey Merz, Carl J. Pepine, Hiroaki Shimokawa, Colin Berry, COVADIS Study Group

**Affiliations:** a British Heart Foundation Glasgow Cardiovascular Research Centre, University of Glasgow, Glasgow, United Kingdom; b Faculty of Medicine, University of Newcastle, Callaghan, Australia; c Department of Cardiology, Gosford Hospital, Central Coast Local Health District, Gosford, Australia; d Department of Cardiology, Robert-Bosch-Krankenhaus, Stuttgart, Germany; e Basil Hetzel Institute, Central Adelaide Local Health Network, University of Adelaide, Adelaide, Australia; f Vita Salute University and San Raffaele Hospital, Milan, Italy; g Department of Cardiovascular and Thoracic Sciences, Fondazione Policlinico A. Gemelli, Università Cattolica del Sacro Cuore, Rome, Italy; h Molecular and Clinical Sciences Research Institute, St. George’s University of London, London, United Kingdom; i Barbra Streisand Women’s Heart Center, Smidt Heart Institute, Cedars-Sinai Medical Center, Los Angeles, California; j Division of Cardiovascular Medicine, University of Florida, Gainesville, Florida; k Department of Cardiovascular Medicine, Tohoku University Graduate School of Medicine, Sendai, Japan; l Department of Cardiology, Golden Jubilee National Hospital, Clydebank, United Kingdom

**Keywords:** angina, ischemic heart disease, microvascular angina, MINOCA, stratified medicine, vasospastic angina, CAD, coronary artery disease, CBF, coronary blood flow, CFR, coronary flow reserve, CI, confidence interval, CTCA, computed tomographic coronary angiography, FFR, fractional flow reserve, IDP, interventional diagnostic procedure, IMR, index of microvascular resistance, INOCA, ischemia with no obstructive coronary artery disease, LV, left ventricular, LVEDP, left ventricular end-diastolic pressure, MACE, major adverse cardiovascular event(s), MB, myocardial bridge, MI, myocardial infarction, MVA, microvascular angina, VSA, vasospastic angina

## Abstract

Ischemic heart disease secondary to coronary vascular dysfunction causes angina and impairs quality of life and prognosis. About one-half of patients with symptoms and signs of ischemia turn out not to have obstructive coronary artery disease, and coronary vascular dysfunction may be relevant. Adjunctive tests of coronary vasomotion include guidewire-based techniques with adenosine and reactivity testing, typically by intracoronary infusion of acetylcholine. The CorMicA (Coronary Microvascular Angina) trial provided evidence that routine management guided by an interventional diagnostic procedure and stratified therapy improves angina and quality of life in patients with angina but no obstructive coronary artery disease. In this paper, the COVADIS study group provide a comprehensive review of why, how, and when coronary vascular dysfunction should be assessed invasively. They discuss the rationale through a shared understanding of vascular pathophysiology and clinical evidence. They propose a consensus approach to how an interventional diagnostic procedure is performed with focus on practical aspects. Finally, the authors discuss the clinical scenarios in patients with stable and acute coronary syndromes in which measurement of coronary vascular function may be helpful for patient care.

## Highlights

• Patients with symptoms of INOCA can have treatable coronary vasomotion disorders.
• Epicardial and microvascular vasospasm may cause MI with no obstructive CAD and type 2 MI.
• Invasive physiological testing (functional coronary angiography) helps exclude, diagnose, and treat these conditions.
• Stratified medicine can transform the management and well-being of these patients.

## Central Illustration

**Figure:**
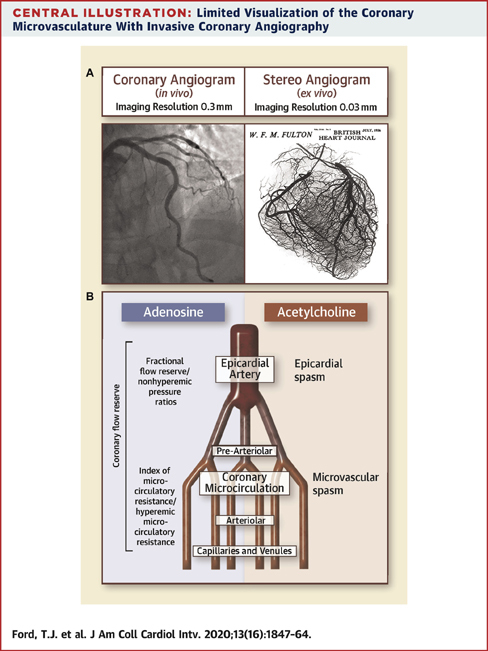

**Figure 1:**
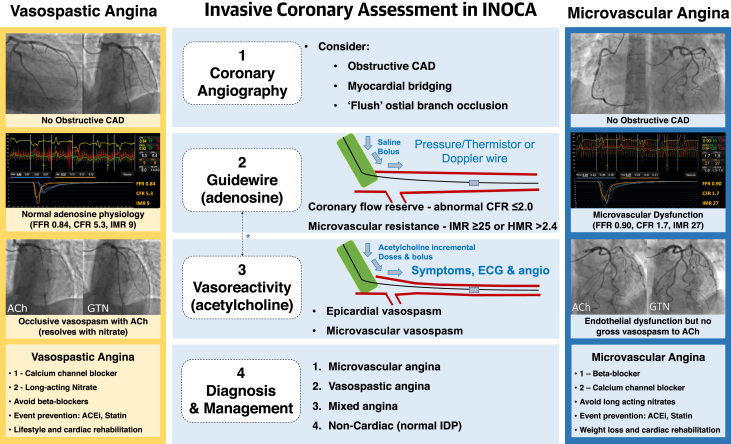
Clinical Utility of an IDP in Patients With Symptoms and/or Signs of Ischemia But No Obstructive CAD Two patients with similar baseline angiograms and clinical presentations without obstructive epicardial coronary artery disease (CAD). Each patient undergoes the an interventional diagnostic procedure (IDP), which reveals a distinct diagnosis. Therapies for microvascular and vasospastic angina are distinct and should be guided by the IDP results. The **yellow figure** shows a typical case of vasospastic angina with preserved microvascular function. The patient was previously on a beta-blocker, and this was substituted for by a calcium-channel blocker with smoking cessation counseling. The **blue figure** depicts a patient with proven microvascular dysfunction but no severe vasospasm. There were abnormalities in both microcirculatory resistance (index of microcirculatory resistance [IMR]) and coronary vasodilator reserve (coronary flow reserve [CFR]). The patient had a diagnosis of microvascular angina and cessation of long-acting nitrate medication with up-titration of a beta-blocker. The patient underwent cardiac rehabilitation classes to assist in weight loss and identify relevant life-style factors implicated in the condition. Note that some operators may prefer to perform vasoreactivity testing before instrumenting the artery for guidewire based invasive CFR and microvascular resistance measurement. ACEi = angiotensin-converting enzyme inhibitor; angio = angiography; DS = diameter stenosis; ECG = electrocardiography; FFR = fractional flow reserve; GTN = glyceryl trinitrate; rehab = rehabilitation.

Ischemic heart disease is a leading global cause of premature disability (1) and death (2). The classic cause of ischemic heart disease is coronary atherosclerosis, but disorders of coronary vasomotion are increasingly recognized (3, 4, 5). Approximately one-half of patients undergoing coronary angiography for known or suspected angina are found to have nonobstructed epicardial coronary arteries, and vasomotion disorders, including microvascular angina (MVA) and/or vasospastic angina (VSA), may be relevant. Coronary angiography has very limited sensitivity for the detection of these disorders (Figure 1).

Epicardial artery spasm causes VSA, first described by Prinzmetal et al. (6) as “variant angina.” Microvascular spasm and/or impaired coronary vasodilation cause MVA, formerly known as cardiac syndrome X (5). Vasospastic disorders of the conduit arteries and microvessels are diagnosed using acetylcholine reactivity testing and often coexist with coronary atherosclerosis. Moreover, coronary vascular dysfunction, whether epicardial or microvascular, can also cause myocardial ischemia in patients with obstructive coronary artery disease (CAD) (3, 4, 5).

Coronary vasomotion disorders cause a relative supply-demand mismatch of myocardial blood flow and nutrients relative to their requirements, inducing myocardial ischemia that may be transient, recurrent, and/or chronic. Ischemia with no obstructive CAD (INOCA) is typically a chronic health problem (7,8). European Society of Cardiology guidelines (9) have revised nomenclature (“chronic coronary syndromes”) in part reflecting the importance of patients with signs and symptoms of INOCA (4,10). When studied using specific tests, MVA and VSA are common findings; up to 4 in 5 patients with INOCA may be affected (11, 12, 13). They are mostly women, and prognosis (14, 15, 16, 17, 18) and quality of life (7,19, 20, 21) are impaired. Vasospasm may also be a primary cause of myocardial infarction (MI) with no obstructive coronary disease and type 2 MI. Although rarely used in daily practice, adjunctive tests of coronary function are supported by emerging clinical trial evidence, and European Society of Cardiology guidelines now support their use (9,11,13,22). Coronary functional disorders also occur among patients with obstructive CAD (3), but current diagnostic testing is limited with an upstream obstructive lesion, so in this review we focus on patients without epicardial obstruction.

In this review, we describe why, how, and when coronary vascular function should be measured in selected patients in the cardiac catheterization laboratory. Interventional cardiologists work at the critical point in the care pathway for diagnosis of and therapy for patients with INOCA, so interventional cardiologists are the target audience for this review. We outline the rationale for why invasive measurements of coronary function are clinically relevant, in line with emerging results from recent trials. We describe how coronary vasomotion assessment with an “interventional diagnostic procedure” (IDP) is performed, with a focus on practical considerations and tips and tricks in the catheterization laboratory. We then describe the clinical indications for when an adjunctive IDP should be performed in daily practice. Finally, we consider future directions.

## Why Measure Coronary Vascular Function?

The rationale for adjunctive testing of coronary vascular function during invasive angiography is 3-fold: diagnosis, prognosis, and treatment implications. First, a normal angiographic study does not exclude a disorder of coronary vascular function. In a symptomatic patient with INOCA, coronary angiography may be considered incomplete without adjunctive diagnostic tests of coronary vascular dysfunction (Central Illustration, Table 1) (9,23,24). Other methods, such as intravascular imaging, are informative for myocardial bridging but not for vascular dysfunction.

**Central Illustration:**
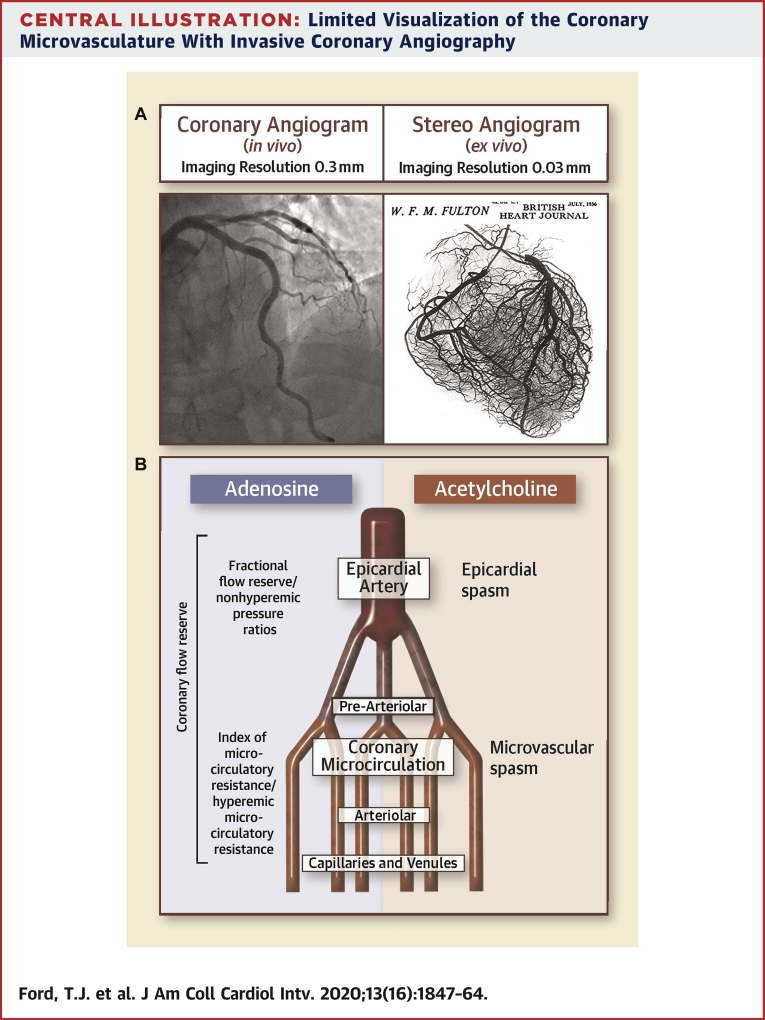
Limited Visualization of the Coronary Microvasculature With Invasive Coronary Angiography **(A)** This figure illustrates a typical normal coronary angiogram **(left)** with a smooth and well-opacified left anterior descending coronary artery. The **right image** is a bismuth stereo angiogram from a cadaveric heart in work performed more than 50 years ago by the late Prof. S. Fulton (reproduced with permission from Fulton [30]). This image offers an unsurpassed illustration of the coronary microcirculation, contrasting starkly with the lack of microcirculatory information on the invasive coronary angiogram (30). **(B)** This schematic illustrates compartmentalized physiological assessment according to the probes acetylcholine and adenosine. The metrics fractional flow reserve and nonhyperemic pressure ratios are predominantly tests of epicardial coronary obstruction to blood flow, whereas index of microcirculatory resistance and hyperemic microcirculatory resistance are more specific to the microcirculatory function. Finally, coronary flow reserve is a metric that can be influenced by any combination of epicardial or microvascular disease or changes in resting flow.

**Table 1:** Proposed Standardized Diagnostic Criteria: Coronary Vascular Dysfunction

Diagnostic Group	Outcome Definitions: Disorders of Coronary Artery Function	
Microvascular angina	• Abnormal microvascular resistance • Impaired coronary vasorelaxation • Microvascular spasm	IMR ≥25 HMR >2.4 CFR by thermodilution <2.0
		Angina during intracoronary infusion of acetylcholine with typical ischemic ST-segment changes without epicardial coronary constriction (<90% reduction) in coronary artery diameter∗
Vasospastic angina	Epicardial spasm	Reduction in coronary diameter >90% following intracoronary acetylcholine from baseline in any epicardial coronary artery segment together with symptoms and ST-segment deviation on ECG†
Obstructive epicardial coronary disease		FFR ≤0.80 Contrast FFR ≤0.83 Resting indices (i.e., iFR, NHPR) ≤0.89
Endothelial dysfunction		Impaired vasodilatation and/or impaired increase in coronary flow velocity in response to intracoronary infusion of low doses (1–30 μg) of acetylcholine

CFR = coronary flow reserve; ECG = electrocardiography; FFR = fractional flow reserve; HMR = hyperemic microvascular resistance; iFR = instantaneous wave-free ratio; IMR = index of microcirculatory resistance; NHPR = nonhyperemic pressure ratio.

∗ Microvascular spasm may occur earlier than diffuse, distal spasm of a conduit artery.

† Prinzmetal vasospasm is typically focal.

Second, in an undifferentiated population of patients undergoing invasive management during daily practice, an IDP empowers cardiologists to make the correct diagnosis with linked therapy (Figure 1). Stratified medicine is the identification of key subgroups of patients (endotypes) within an undifferentiated, heterogeneous population, these endotypes (MVA, VSA, both, or none) being distinguishable by distinct mechanisms of disease and/or responses to linked therapy (Figure 1) (25). The tests empower clinicians to include or exclude coronary vascular dysfunction in affected patients, and discrimination of angina due to vasospasm and/or impaired vasodilator reserve (functional disorder) from increased microvascular resistance (structural disorder) permits specific and distinct treatments outlined in practice guidelines (9).

Third, demonstration of coronary vascular dysfunction as a mechanism or cause of myocardial ischemia provides new prognostic information empowering patients and clinicians to adopt optimal guideline-directed preventive therapy and enhancing treatment satisfaction (14,16).

### Diagnosis

Coronary angiography is the standard-of-care test for identifying obstructive CAD either by anatomic imaging using noninvasive computed tomographic coronary angiography (CTCA) or invasive coronary angiography (26,27). Although procedure numbers worldwide are uncertain, approximately 10 million invasive coronary angiographic examinations are performed annually, including 4 million per year in Europe and the United States (28,29). Invasive coronary angiography has a spatial resolution of approximately 0.5 mm, and evaluation is determined by subjective visual interpretation. The limited spatial resolution of angiography does not allow visualization of the resistance arterioles (20 to 400 μm) that largely govern myocardial blood flow (Figure 1) (30).

The noninvasive management of symptomatic patients has evolved in recent years. In Europe, practice guidelines for the management of symptomatic patients with high (>85%) pre-test probability of a coronary artery stenosis support direct referral for invasive coronary angiography with or without a functional assessment with either a nonhyperemic pressure ratio or fractional flow reserve (FFR) (9). Noninvasive CTCA is associated with high sensitivity for the detection of epicardial CAD. In the United Kingdom, Clinical Guideline 95 of the National Institute for Health and Care Excellence recommends CTCA as the first-line diagnostic technique for patients with anginal chest pain and no history of CAD (31). Thus, an increasing proportion of patients who undergo invasive coronary angiography have not undergone functional stress testing, meaning that information on ischemia is often lacking at the time of anatomic testing with either invasive or noninvasive angiography. This gap presents new challenges for decision making in patients with INOCA. Practice guidelines recommend (Class I, Level of Evidence: A) the use of invasive measures of coronary disease severity to assess for flow-limiting coronary disease (32,33), but many consensus guidelines do not emphasize invasive testing of coronary vascular function in patients with INOCA. This means that clinicians do not assess for ischemia caused by disorders of coronary vasomotion, leading to diagnostic uncertainty.

Looking forward, coronary microvascular dysfunction presents an unmet therapeutic need, and novel therapies, including implantable devices, are being actively pursued. Examples include the coronary sinus reducer stent for the treatment of refractory angina (NCT02710435) and pressure-controlled intermittent coronary sinus occlusion in acute MI (NCT03625869). The main objective of coronary sinus device therapy is to induce a controlled increase in coronary sinus blood pressure, thereby increasing retrograde myocardial perfusion to reduce the propensity to myocardial ischemia. Clinical evidence from randomized controlled trials involving coronary sinus device therapy is awaited with great interest.

### Prognosis

Patients with undiagnosed chest pain (including those who have undergone cardiac investigations) are at increased risk for cardiovascular events for at least 5 years (34). Women with angina appear to be particularly burdened by symptoms and morbidity even after reassuringly “normal” findings on invasive coronary angiography (35).

There are evolving data from many large prospective studies on the independent prognostic impact of coronary microvascular disease on major adverse cardiovascular event(s) (MACE). Data from the National Heart, Lung, and Blood Institute–sponsored WISE (Women’s Ischemia Syndrome Evaluation) study suggest that there is a worse prognosis in patients with INOCA: the 5-year annualized risk for MACE was 16.0% in women with nonobstructive CAD, 7.9% in women with normal coronary arteries, and 2.4% in an asymptomatic control group (p ≤ 0.002 after adjustment for baseline cardiovascular risk) (36). After mean follow-up of 5.4 years, the time-to-event analysis confirmed that low coronary flow reserve (CFR) was a robust independent predictor of MACE (hazard ratio: 1.20; 95% confidence interval [CI]: 1.05 to 1.38; p = 0.008). Similarly, a large Danish cohort study of 11,223 patients found an increased risk for MACE among patients with angina with diffuse nonobstructive CAD and those with normal coronary arteries (adjusted hazard ratios: 1.85 and 1.52, respectively), compared with a reference population. Taqueti et al. (14) recently produced a provocative study showing that MACE risk in women is driven by reduced CFR and not obstructive CAD, with CFR an important predictor of events even in those without obstructive CAD (adjusted hazard ratio: 1.69; 95% CI: 1.04 to 2.76; p = 0.03) (14). The adverse prognostic importance of impaired coronary vasomotion has also been identified in a meta-analysis of 6 studies including 1,192 subjects who experienced 243 cardiovascular events during a follow-up period of 3.8 to 9.7 years. The overall relative risk was 2.38 (95% CI: 1.74 to 3.25), and the risk (2.49) was even higher in 1,048 patients (n = 209 events) who had undergone acetylcholine reactivity testing (37).

### Treatment

Historically, there was no randomized evidence that a diagnostic strategy linked to therapy improves patient well-being. The CorMicA (Coronary Microvascular Angina) trial was undertaken to address this evidence gap (11,38). Patients with INOCA were randomized 1:1 to the intervention group (stratified medical therapy, interventional or functional diagnostic procedure disclosed) or the control group (standard care, IDP performed, results not disclosed). The diagnosis of a clinical endotype (MVA, VSA, both, or none) was linked to guideline-based management (10). After disclosure of the IDP result, more than one-half of treating clinicians changed the initial diagnosis and treatment on the basis of angiography alone. The intervention was associated with a mean improvement of 11.7 units in the Seattle Angina Questionnaire summary score [11] at 6 months (95% CI: 5.0 to 18.4; p = 0.001) (the primary endpoint) associated with improvements in quality of life (EQ5D index: 0.10 U; 95% CI: 0.01 to 0.18; p = 0.024). Longer term follow-up to 1 year has confirmed that these benefits are maintained (39). In summary, the CorMicA study provides clinical evidence of better quality of life for patients with angina without obstructive CAD when management is guided by invasive tests of coronary vascular function.

### Therapeutic nihilism and sex bias?

Some clinicians may take the view that patient benefits can be achieved by assessing coronary function (40). A simpler, pragmatic approach may be to administer a trial of medical therapy as a matter of routine in all symptomatic patients and assess their responses over time, representing a trial of therapy. An angiography-guided approach avoids prolonging the procedure (about 15 min) and the cost (guidewire, adenosine, and acetylcholine) of the IDP. We contend that therapeutic nihilism is not in the best interest of patients and that precision medicine (the right treatment for the right patient at the right time) is preferred (25). This may be especially relevant considering that affected patients are often women (41). Practice guidelines give clear treatment protocols for these conditions (9), now supported by evidence from randomized, controlled trials. Furthermore, avoiding unnecessary medicines and optimizing therapy when linked to the correct diagnosis will benefit patients, health care providers, and the health care system (11).

### Coronary physiology and diagnosis of vasomotor disorders

Coronary vascular function reflects contributions from the epicardial conduit coronary arteries, its intramyocardial branches, and the microcirculation. The key functional parameters are vascular tone, vasodilator reserve, and resistance. Coronary resistance is determined mainly by intramural arterioles <400 μm in diameter. CFR reflects the vasodilator capacity of the coronary circulation. CFR is a global measure of vasodilator capacity that may be impaired by abnormalities of the conduit coronary arteries, the microcirculation through to the capillaries, or both compartments. CFR may also be limited if basal flow is high, if diastolic time is reduced, or if intramyocardial pressure is increased (42).

### Pathophysiologic basis of coronary vasomotor disorders

A disorder of coronary vascular function can be caused by structural and/or functional abnormalities (3, 4, 5), and the vasodilator response to hyperemic stimulants, such as pharmacological stress (43,44) or exercise (45), may be impaired. Coronary microvascular dysfunction (increased resistance) may result from remodeling of the vascular wall, inflammation, alterations in the composition and volume of the extravascular (interstitial) matrix (46), and systemic changes including capillary rarefaction (47) and arteriolar dysfunction (48, 49, 50).

Vascular function may vary among different coronary artery territories, and normal global CFR may mask impaired vasodilator reserve in a single major artery (42). Regional differences and variations in resting flow support the rationale for estimation of coronary flow capacity by positron emission tomography (49) and for assessing multiple coronary arteries during invasive management, when clinically appropriate.

Coronary artery spasm represents acute, flow-limiting vasoconstriction (51). Kaski et al. (52) showed that coronary hyperreactivity is responsible mainly for focal rather than diffuse epicardial vasospasm. Coronary artery spasm is caused by hyperreactivity of vascular smooth muscle cells and a triggering stimulus. The cause of vascular smooth muscle cell hyperreactivity is incompletely understood. Endothelial dysfunction is associated with coronary artery spasm, enhancing its likelihood and severity, but endothelial dysfunction is not the primary driver (51). Cardiovascular risk factors, inflammation, oxidative stress, genetic factors, and ethnic differences are implicated. Coronary artery imaging using ^18^F-fluorodeoxyglucose positron emission tomography/computed tomography has identified localized inflammation in the coronary adventitia and perivascular adipose tissue of patients with VSA (53). Rho-kinase mediates epicardial coronary spasm and microvascular spasm, especially in patients with microvascular dysfunction (54). Autonomic imbalance, hyperventilation, and platelet activation are potential triggers. Ethnic differences in coronary spasm, such as in Japanese patients (53), reflect an expansion of the personalized medicine concept.

Endothelial dysfunction typically precedes and causes atherosclerosis. Endothelium-derived nitric oxide mainly mediates vasodilatation of the conduit epicardial coronary arteries, whereas endothelium-derived hyperpolarizing factors–mediated responses determine endothelium-dependent vasodilatation of resistance arteries (e.g., coronary microvessels) (55). Endothelial dysfunction is associated with vascular risk factors, including diabetes mellitus and circulating inhibitors of nitric oxide synthase, as reflected by serum concentrations of asymmetrical dimethylarginine (13,56), and low endothelial shear stress (57). The pathophysiology of endothelial dysfunction (e.g., redox imbalance) is distinct from vasospasm (rho-kinase-induced myosin light chain phosphorylation) (55). Coronary endothelial dysfunction is therapeutic target for lifestyle and pharmacological interventions, notably statins and angiotensin-converting enzyme inhibitors.

Endothelial function of the coronary artery may be defined according to the method used. If assessed using coronary angiography, endothelial dysfunction is defined as a decrease in 1 or more segments of an epicardial coronary artery luminal diameter of >20% after intracoronary infusion of low doses of acetylcholine (58). Normal endothelial function may be defined as normal (%Δ coronary artery diameter [acetylcholine] >20%), mild endothelial dysfunction (%Δ diameter [acetylcholine] 20% to −20%), or severe endothelial dysfunction (%Δ coronary diameter [acetylcholine] <−20%) (59). Endothelium-dependent epicardial vasomotion can also be assessed by calculating the percentage in coronary cross-sectional area change in response to intracoronary acetylcholine (change in epicardial cross-sectional area >0% is considered normal) (16).

Endothelial dysfunction may also be described according to changes in coronary blood flow (CBF) in response to infusion of acetylcholine (16,59). Normal coronary endothelium-dependent function is defined as a Doppler-derived increase in CBF of ≥50% (i.e., a ratio of >1.5 in response to acetylcholine, calculated by dividing CBF after 10^−4^ mol/l acetylcholine [18.2 μg/ml] by the baseline). Endothelial dysfunction can be further classified as mild (0% to <50% change in CBF) or severe (<0% change in CBF). Impaired coronary endothelium-independent function can be defined as a ratio of flow velocity to adenosine, with cutoffs varying from ≤2.0 to 2.5 (16,60).

Coronary endothelial dysfunction revealed by acetylcholine reactivity testing in the catheterization laboratory is associated with inducible myocardial ischemia determined by injection of ^99m^Tc sestamibi and single-photon emission computed tomography (61). In a study of 299 patients undergoing coronary angiography and endothelial function testing, 60 had normal endothelial function and 239 had abnormal endothelial function. When stratifying patients by the presence or absence of endothelial dysfunction, in those with preserved endothelial function, troponin I concentrations were higher in patients who developed MACE during 7.0 ± 0.3 years of follow-up compared with those who did not (1.35 ng/l [interquartile range: 1.1 to 2.1 ng/l] vs. 0.7 ng/l [interquartile range: 0.7 to 1.1 ng/l]; p = 0.02) (62). These findings are important because coronary endothelial dysfunction is a modifiable, therapeutic target for life-style interventions and medical therapy (statins, angiotensin-converting enzyme inhibitors), and a clinical strategy based on endothelial function testing may improve quality of life (22). Two trials of endothelin-1 receptor antagonists in patients with MVA reported favorable results (63,64). The potential for patient benefits with endothelin receptor antagonist therapy is currently being evaluated in a precision medicine trial of zibotentan in MVA (NCT04097314 and ACTRN12618000021279).

## How to Assess Coronary Vascular Dysfunction in the Catheterization Laboratory?

### Setup

**Figure 2:**
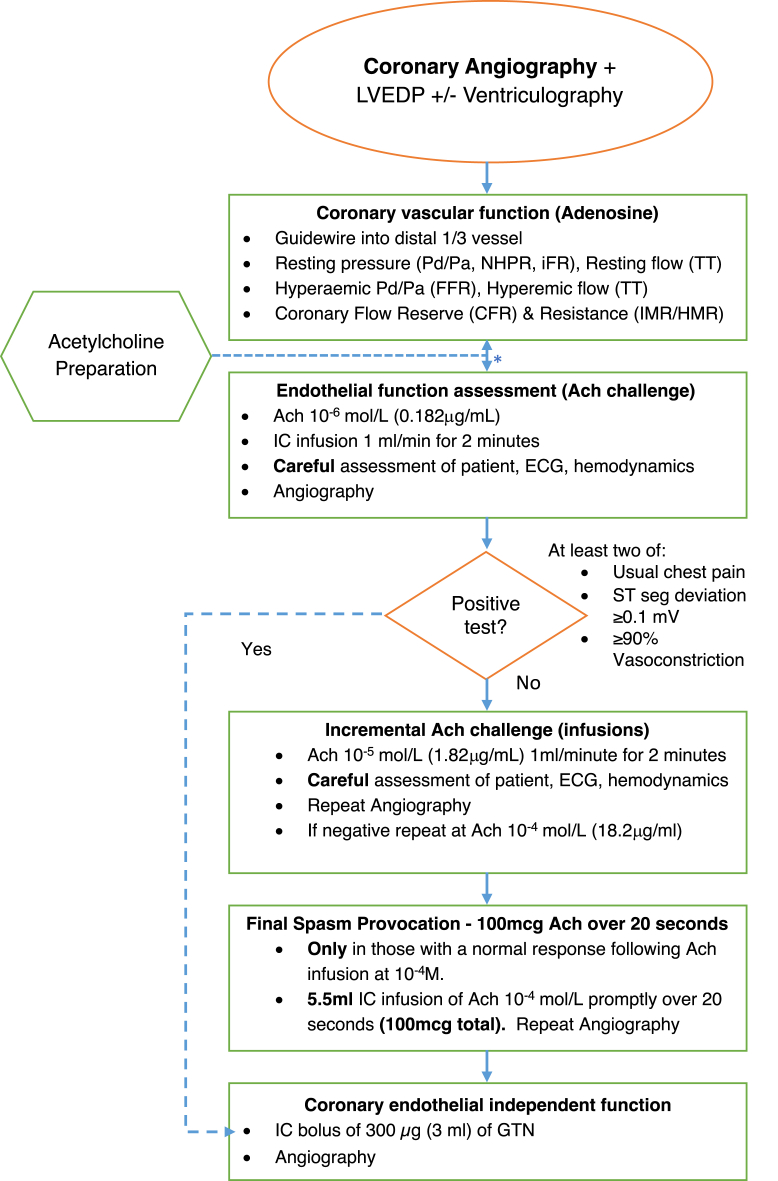
Cardiac Catheterization Laboratory Interventional Diagnostic Procedure Protocol Proposed step-by-step approach to guidewire-based assessment of coronary vascular function using thermodilution or Doppler and then vasoreactivity testing using acetylcholine (Ach). This simple approach focuses on thermodilution, which is straightforward to include during daily practice. Note that some operators may prefer to perform vasoreactivity testing first without the guidewire, allowing Ach challenge prior to any short-acting nitrate administration. HMR = hyperemia microvascular resistance; IC = intracardiac; LVEDP = left ventricular end-diastolic pressure; LV gram = left ventriculogram; NHPR = nonhyperemic pressure ratio; seg = segment; TT = transit time (for bolus of normal saline); other abbreviations as in Figure 1.

The purpose of this section is to give practical guidance to clinicians on how to assess coronary vascular function in the catheterization laboratory (Table 2) (65,66). A step-by-step guide is illustrated in Figure 2. Vasoactive medications should be withheld for at least 24 h. Coronary vascular function can be assessed by a trained cardiologist using invasive techniques. Radial artery access generally works well. A cocktail of intra-arterial vasodilator drugs to prevent radial artery spasm may confound subsequent measurements of coronary function. We generally avoid the use of intra-arterial calcium channel antagonists and longer acting nitrates (e.g., verapamil and isosorbide dinitrate). Glyceryl trinitrate has a short half-life and is preferred. Standard coronary catheters can be used, although the benefits of a smaller arteriotomy and guide catheters (5-F) include reduced radial spasm and reduced risk for vascular injury.

**Table 2:** Practical Considerations for Invasive Assessment of Coronary Vascular Function

Procedure	Practical Points
Set-up	Acetylcholine may be pre-ordered, according to local arrangements.
	Obtain informed consent.
	Undertake team briefing on indication and protocol.
	Administer heparin 5,000 IU (as per local standard care procedures).
	Use radial artery access; avoid administration of vasodilator drugs, as they may confound measurement of coronary vascular function.
	Administer short-acting intra-arterial GTN (avoid verapamil/GDN).
	Use a 5-F guide catheter to reduce spasm in small radial arteries.
Coronary angiography	
Projection	Choose an imaging projection that reveals the long axis of the target vessel (i.e., no foreshortening), with minimal vessel overlap.
TIMI frame count	Ensure that cine acquisition is sufficiently long to assess for myocardial blush of contrast media.
Diagnostic guidewire	A single target coronary artery may be sufficient for diagnosis and decision making; in general, select the left anterior descending coronary artery.
	If normal results are obtained and clinical suspicion remains high, consider undertaking the IDP in a second coronary artery.
	Advance the guidewire into the distal third of the target coronary artery.
ComboWire Doppler	Consider using a buddy wire to safely advance the ComboWire.
Coronary reactivity testing	Avoid a vasodilator cocktail in radial procedures.
	Retain the buddy wire in situ to facilitate direct intracoronary testing.
	A dedicated intracoronary catheter is generally not necessary (and may increase the risks of the procedure); injection of acetylcholine is done through the guiding catheter into the lumen of the left main coronary artery. Prior to starting the infusion of acetylcholine, initially flush the lumen of the guide with ∼2 ml of the infusate (depending on the French size of the catheter used) to replace the flushing saline in the shaft of the catheter. Once the acetylcholine solution has reached the tip of the catheter, further injection is done more slowly and steadily over 20 s. The catheter is then slowly refilled with saline, remembering that this procedure will lead to extrusion of acetylcholine at the tip of the catheter for at least as long until all the acetylcholine solution is replaced by saline.
	If infusing into a “dominant” coronary artery, consider “half dose” of the acetylcholine to limit bradycardia.
	In cases with normal coronary function or “negative” test responses, if clinical suspicion persists, a dose of 200 μg may be infused into the left coronary artery, increasing sensitivity without impairment of specificity.
	Use isosorbide dinitrate, which has short-acting effects, unlike GTN.

GDN = glyceryl dinitrate; GTN = glyceryl trinitrate; IDP = interventional diagnostic procedure.

### Coronary angiography

The cardiologist visually assesses antegrade flow of contrast media during cine angiography. Semiquantitative analysis may be undertaken by calculating the TIMI (Thrombolysis In Myocardial Infarction) frame count (49). In patients with unobstructed epicardial coronary arteries, a corrected TIMI frame count >27 (images acquired at 30 frames/s) suggests MVA due to impaired resting flow (coronary slow-flow phenomenon) (67). Slow flow points to an increase in vascular resistance under resting conditions and is typically seen in male smokers and may be implicated in propensity to acute coronary syndromes (68).

We typically use a diagnostic JR4 (Judkins right) catheter to perform angiography of the right coronary artery before crossing the aortic valve to measure left ventricular end-diastolic pressure (LVEDP). Elevated LVEDP may reflect heart failure, which is associated with CMD (69). We then use a left coronary guiding catheter with reasonable support (e.g., EBU3.5, extra backup), which permits reproducible transit time injections and good intubation of the left main coronary artery for acetylcholine infusions. In choosing a guiding catheter, we exercise caution to ensure coaxial coronary intubation and avoidance of pressure damping to avoid injury of the vascular wall.

### Interventional Diagnostic Procedure (IDP)

The IDP is a combinatory technique involving direct invasive measurements of coronary vascular function initially with a diagnostic guidewire, then acetylcholine reactivity testing (Tables 1 and 2, Figure 1). As a practical guide, following acquisition of the coronary angiogram, we recommend that the IDP initially focus on the use of a diagnostic guidewire and then, as appropriate and feasible, acetylcholine vasoreactivity testing. There is no firm consensus on the approach (9,60). We advocate this diagnostic sequence because, should vasospasm occur following intracoronary infusion of acetylcholine, the assessment of resting physiology becomes confounded by elevated sympathetic drive. An alternative approach sees vasospasm provocation first, before assessment of CFR. This approach is advocated by some who have concerns about coronary vasospasm testing if a short-acting nitrate is initially administered (e.g., to prevent vasospasm if using radial artery access or to optimize coronary angiography). In the authors’ experience, epicardial coronary spasm is still readily provoked despite nitrate administration during transradial access for angiography.

### Diagnostic guidewire

The guidewire procedure is performed as an adjunct to coronary angiography. The IDP should be focused to a single major coronary artery to limit the duration of the procedure. Additional studies in a second coronary artery may be appropriate if the initial test results are negative and clinical suspicion is high.

The left anterior descending coronary artery is usually preferred as the pre-specified target vessel, reflecting its subtended myocardial mass and coronary dominance (Table 2), and this artery is in our experience typically reactive to the effects of acetylcholine. If technical factors, such as tortuous coronary anatomy, preclude instrumentation of this artery, then the circumflex or right coronary artery should be assessed. Intravenous heparin (50 to 70 U/kg) should be administered to achieve therapeutic anticoagulation (activated clotting time ∼250 s) before coronary instrumentation. Diagnostic options include coronary thermodilution using a pressure-temperature sensor guidewire (PressureWire X, Abbott Vascular, Santa Clara, California) or a Doppler technique (ComboWire XT or Flowire, Philips Volcano Corporation, San Diego, California). The ComboWire XT connects to the ComboMap system (Philips Medical Systems, Eindhoven, the Netherlands).

Typically, intra-arterial glyceryl trinitrate is given routinely during coronary angiography, although we suggest using 200 μg or less. The half-life of glyceryl trinitrate is about 2 min, and thus after 10 min, only 3% of the medication is active, so it is therefore unlikely to suppress a false-positive result for epicardial vasospasm (70). The usual approach to inducing steady-state hyperemia is by use of intravenous adenosine (140 μg/kg/min) administered through a large peripheral vein. Intravenous adenosine activates vascular A2 receptors, leading to predominantly non-endothelium-dependent vasodilation, although there may also be a lesser component of endothelial-dependent vasodilation (71). Intracoronary bolus injection of adenosine (up to 200 μg) or nicorandil (2 mg) is an alternative option to assess endothelium-independent vasodilatation (40). The adenosine infusion is given for 2 to 3 min, and although mild symptoms are common, it is generally well tolerated. Hemodynamic markers of coronary hyperemia are: 1) “ventricularization” of the distal pressure waveform; 2) disappearance of distal dicrotic pressure notch; and 3) separation of mean aortic and distal pressures (72). Changes in heart rate, blood pressure, and rate-pressure product are less reliable measures of coronary hyperemia (73).

### Coronary thermodilution

The principle of coronary thermodilution is that transit time, derived from a bolus intracoronary injectate of normal saline administered at room temperature to mix with blood at body temperature, represents the inverse of CBF (74). From a practical perspective, the diagnostic guidewire connects wirelessly to transmit data to a personal computer using dedicated analysis software (Coroventis, Uppsala, Sweden). The guidewire sensor tip is positioned at the tip of the guiding catheter, and the pressure measurement from the wire is equalized with that of the guiding catheter. The guiding catheter should be coaxial with the long axis of the coronary artery to ensure effective delivery and mixing of saline. The sensor is then positioned in the distal third of the coronary artery followed by 3 intracoronary injections of saline (3 ml) at room temperature. The mean transit time is measured with each bolus and averaged to calculate the resting mean transit time. When steady-state hyperemia is achieved by pharmacological stress testing, 3 additional injections of 3 ml of room-temperature saline are performed. The transit time is automatically measured after each set of injections and averaged to calculate the hyperemic mean transit time. Simultaneous measurements of mean aortic pressure (by guiding catheter) and mean distal coronary pressure (by pressure wire) are also made during maximal hyperemia.

CFR is calculated using thermodilution as resting mean transit time divided by hyperemic mean transit time (abnormal CFR is defined as ≤2.0) (60,75). The index of microvascular resistance (IMR) is calculated as the product of distal coronary pressure at maximal hyperemia multiplied by the hyperemic mean transit time (76). IMR has a weak correlation with the subtended myocardial mass, leading some to propose vessel-specific cutoffs. A guiding catheter must also be intubated well within the left main coronary artery to ensure reproducible coronary transit time estimates. The normal values for IMR and CFR have been challenging to define. The normal range of IMR is considered to be <25, on the basis of 3 studies evaluating IMR in different populations (77, 78, 79, 80). The only truly “healthy” population used to validate IMR was 20 subjects who underwent IMR testing prior to ablation for supraventricular rhythm disturbance. In this study, Solberg et al. (80) noted the upper limit of the estimated 95th percentile for IMR in 20 healthy control subjects to be 27 (95% CI: 21 to 34). The investigators stated that if a larger cohort of control subjects were used, this upper limit would likely be reduced. More recently, IMR ≥18 was identified as the optimal cutoff for the prediction of MACE in an Asian population of subjects with INOCA (54).

Flow-limiting coronary disease may be calculated during the same setting of adenosine-induced hyperemia simultaneously from the ratio of mean distal coronary pressure to mean aortic pressure at maximal hyperemia; abnormal FFR is defined as ≤0.80 (33) or a nonhyperemic pressure ratio (81). We advocate a patient-centered approach to decision making. The binary thresholds of continuous data should be viewed within the context of the patient. A CFR between 2.0 and 2.5 reflects an impaired vasodilator reserve and may be considered a CFR “gray zone,” as is also the case for FFR (0.75 to 0.82). CFR, IMR, nonhyperemic pressure ratio, and FFR have prognostic significance across the diagnostic range of their values. An accepted caveat of CFR measured by any modality is its inherent variability related to influence of resting hemodynamic status. CFR is also affected by epicardial CAD and so is not specific to microcirculatory pathology.

### Pressure and flow measurements

The relative simplicity and accessibility of thermodilution-derived CFR and IMR are attractive, but there are inherent limitations. The setup conditions should be constant during the thermodilution measurements. Specifically, the guide catheter should be engaged without pressure damping, and the position of the guidewire sensor should be constant to reduce variability in the saline transit times. Coronary vascular function can be assessed using a pressure-flow wire (ComboWire XT) or a Doppler wire to measure coronary flow velocity (Flowire) (66). CFR assessed using thermodilution (82) slightly overestimates flow reserve at higher levels compared with CFR assessed using Doppler. Doppler-derived hyperemic microvascular resistance may be a closer correlate of microvascular function assessed noninvasively using cardiac magnetic resonance (myocardial perfusion reserve) (83). Simultaneous measurement of coronary flow velocity reserve with pressure enables myocardial resistance (hyperemic microvascular resistance) to be calculated (84).

Selective intracoronary infusion of acetylcholine using a dedicated microcatheter may be preferred rather than infusing the acetylcholine through a guiding catheter (66). The advantage of using a microcatheter is the subselective infusion of acetylcholine and, potentially, avoidance of pancoronary vasospasm. The disadvantage of this approach is the additional coronary instrumentation, related risks for vascular injury, and expense. We think there are pros and cons to using a microcatheter. In the end, operator preference and the diagnostic circumstances of the procedure should guide the approach on an individual patient basis.

A Doppler wire may be used to measure coronary flow velocity during intracoronary infusion of acetylcholine (22). When using Doppler, the infusion catheter is placed in the proximal segment of the target artery, and the Doppler wire is sited in the mid to distal segment. Because the Doppler wire is less flexible than a standard coronary guidewire, a “buddy wire” or a microcatheter may be needed to safely advance the Doppler wire into the target artery. Coronary angiography is acquired to estimate the diameter of the coronary artery at baseline and after each infusion of acetylcholine. A projection without foreshortening is essential.

### Pharmacological coronary reactivity testing in the catheterization laboratory

Coronary vascular function is assessed by infusion of a vasoactive substance such as acetylcholine, substance P, or ergonovine (65). The physiological alterations to vascular tone following intracoronary infusion of these substances are determined by the relative functions of the endothelium and smooth muscle cells (85). Vasodilatation reflects a dominant response mediated by endothelial cells (vascular health) over the constrictor effects of vascular smooth muscle cells, whereas vasoconstriction reflects a dominant smooth muscle cell effect over impaired endothelial cell–mediated vasorelaxation (vascular dysfunction).

The most established approach for vasoreactivity testing is by intracoronary infusion of acetylcholine (60). We support a pragmatic approach for coronary reactivity testing according to whichever protocol might work at individual centers. A standard approach involves sequential infusion of acetylcholine at concentrations approximating 0.182, 1.82, and 18.2 μg/ml (10^−6^, 10^−5^, and 10^−4^ mol/l, respectively) at 1 ml/min for 2 min using a mechanical pump. These doses were historically derived using experiments adopting subselective infusion through an intracoronary catheter into the left anterior descending coronary artery, assuming a resting flow rate of 80 ml/min. The effective concentration of acetylcholine at the tissue level was estimated at 10^−8^ to 10^−6^ M. Alternative options to facilitate ease of adoption include manual infusion of 2, 20, 100, and 200 μg (86). Susceptibility to coronary vasospasm is assessed by manual infusion of 100 μg (5.5 ml 10^−4^ M) or 200 μg (11 ml 10^−4^ M) over 20 s into the left main coronary artery (87). On a case-by-case basis, a dose of 200 μg may be infused to enhance sensitivity without adversely affecting specificity (86,88).

When microvascular spasm occurs, coronary flow transiently reduces or ceases in the absence of epicardial coronary artery spasm; that is, the diameter of the coronary diameter is maintained in association with transient reduction of flow (TIMI flow grade ≤2), while the patient generally experiences chest pain in association with ischemic changes on electrocardiography. Prompt recovery is typical, and nitrates can be administered if necessary. Epicardial coronary spasm is defined according to the COVADIS (Coronary Vasomotion Disorders International Study) criteria requiring reproduction of chest pain and ischemic electrocardiographic changes in association with ≥90% vasoconstriction (89). In the case of severe epicardial spasm, it may not be possible to determine whether microvascular spasm coexists. Reflecting the role of the right coronary artery to supply the sinus and atrioventricular nodes (or circumflex in left-dominant anatomy), transient bradycardia commonly occurs. Given the propensity of acetylcholine to induce bradycardia, safety is ensured by administering a half dose (i.e., 50 μg instead of 100 μg). Historically, a temporary implantable transvenous pacing line was used to balance this risk, but this procedure is not without risk and, in our view, is not routinely needed, unless the right coronary artery is infused. We advocate proceeding without transvenous pacing and applying caution with testing the right coronary or left-dominant circulations. Self-limiting atrial fibrillation is also common (8%), particularly during evaluation of right coronary vascular function (90). Patients undergoing clinically indicated coronary angiography typically have risk factors for cardiovascular disease, including atrial fibrillation.

Ergonovine may induce coronary vasospasm via serotonin 1D receptors on vascular smooth muscle cells. Intracoronary ergonovine (20 to 60 μg) is an alternative to acetylcholine for the assessment of coronary vasospasm in some Asian countries (91). Acetylcholine is useful for assessing macrovascular and microvascular function, is safer, and is more widely available.

### Intramyocardial coronary course: Invasive pharmacological assessment

Myocardial bridging is also prevalent in INOCA, probably because of endothelial dysfunction within and distal to affected segments (92). Coronary reactivity testing in patients using a myocardial bridge (MB) may provoke transient spasm and chest pain that reproduces their symptoms. Furthermore, the ischemia-generating potential of MBs may have contributions from dynamic epicardial coronary obstruction. Despite a predominantly systolic effect of MBs, it has been demonstrated that MBs also affect diastolic flow, particularly under enhanced inotropism and tachycardia, both occurring during physical exercise. Inotropic challenge with pressure guidewire interrogation during dobutamine in addition to acetylcholine has diagnostic value in such patients (93).

### Nonpharmacological approaches to stress testing in the catheterization laboratory

Atrial pacing has been used to increase CBF and shear stress in assessing vasoactive responses (94). However, this approach limited because the achievable maximal tachycardia is limited by Wenckebach block, thus affecting CFR determination. Supine exercise testing during coronary angiography with radial or brachial artery access is feasible and can provide clinically relevant information on disease mechanisms (95). Rahman et al. (95) measured coronary flow velocity and pressure under resting conditions, during intravenous adenosine–mediated hyperemia (140 μg/kg/min), and during bicycle exercise using a supine ergometer in the catheterization laboratory. They found that in patients with angina without obstructive CAD (n = 85), CFR but not microvascular resistance identified patients with maladaptive physiological responses to exercise and subendocardial myocardial ischemia (n = 55; hyperemic subendocardial/subepicardial perfusion ratio <1.0, as revealed by stress perfusion cardiovascular magnetic resonance). This finding ties in with the idea of CFR as an invasive functional correlate of impairments in exercise capacity and myocardial perfusion and lends support to the role of exercise testing in the catheterization laboratory.

### Procedural safety

The risks of an IDP are those of coronary instrumentation with a guidewire and adverse physiological reactions. In CorMicA, IDPs were feasible with diagnostic information achieved in 99% of the study population. No serious adverse events occurred. Considering adverse effects of coronary reactivity testing, atrial fibrillation occurred in 1 in 20 patients. This was self-limiting in all but 1 patient, in whom chemical cardioversion was achieved with intravenous amiodarone. Transient bradycardias reflect expected physiological responses, which will resolve immediately after discontinuation of the acetylcholine infusion. A coughing maneuver may be helpful, and vasospasm is typically transient. The cardiac catheterization laboratory environment facilitates patient safety. Multiple publications support the safety of coronary reactivity testing when administered in trained hands (44).

Coronary injury may occur secondary to the guiding catheter or diagnostic guidewire, typically at the start of the standard care procedure. These complications are more likely to occur at the start of the procedure in the hands of an inexperienced trainee when the guiding catheter is less compliant. Rarely, a dissection may be secondary to the diagnostic guidewire. For these reasons, the IDP should be performed by an experienced interventional cardiologist or by a trainee under direct supervision. Coronary dissections are not a consequence of the effects of acetylcholine.

### Complementary measurements

LVEDP is a clinically relevant parameter that is straightforward to measure and provides information on fluid balance and left ventricular (LV) pump function. A low LVEDP (i.e., <3 mm Hg) points to dehydration. Increased LVEDP may reflect volume overload (normal pressure-volume relationship), abnormal LV filling or compliance (diastolic dysfunction), abnormality of LV contractility (systolic dysfunction), or a combination of these factors. We recommend that LVEDP be measured routinely during invasive diagnostic procedures. An indwelling LV catheter may also measure alterations in LVEDP during infusion of acetylcholine. When prior information on LV function is not already available, ventriculography should be considered. Furthermore, depending on the results of left heart catheterization, occasionally, ad hoc right heart catheterization may be appropriate (e.g., to assess for intracardiac shunts, pulmonary hypertension, or as an alternative cause of exercise impairment). Noninvasive imaging using echocardiography and cardiovascular magnetic resonance imaging provides complementary diagnostic information, notably on LV systolic and diastolic function, LV mass, valve function, and pulmonary artery systolic pressure.

## When to Measure Coronary Vascular Function

**Figure 3:**
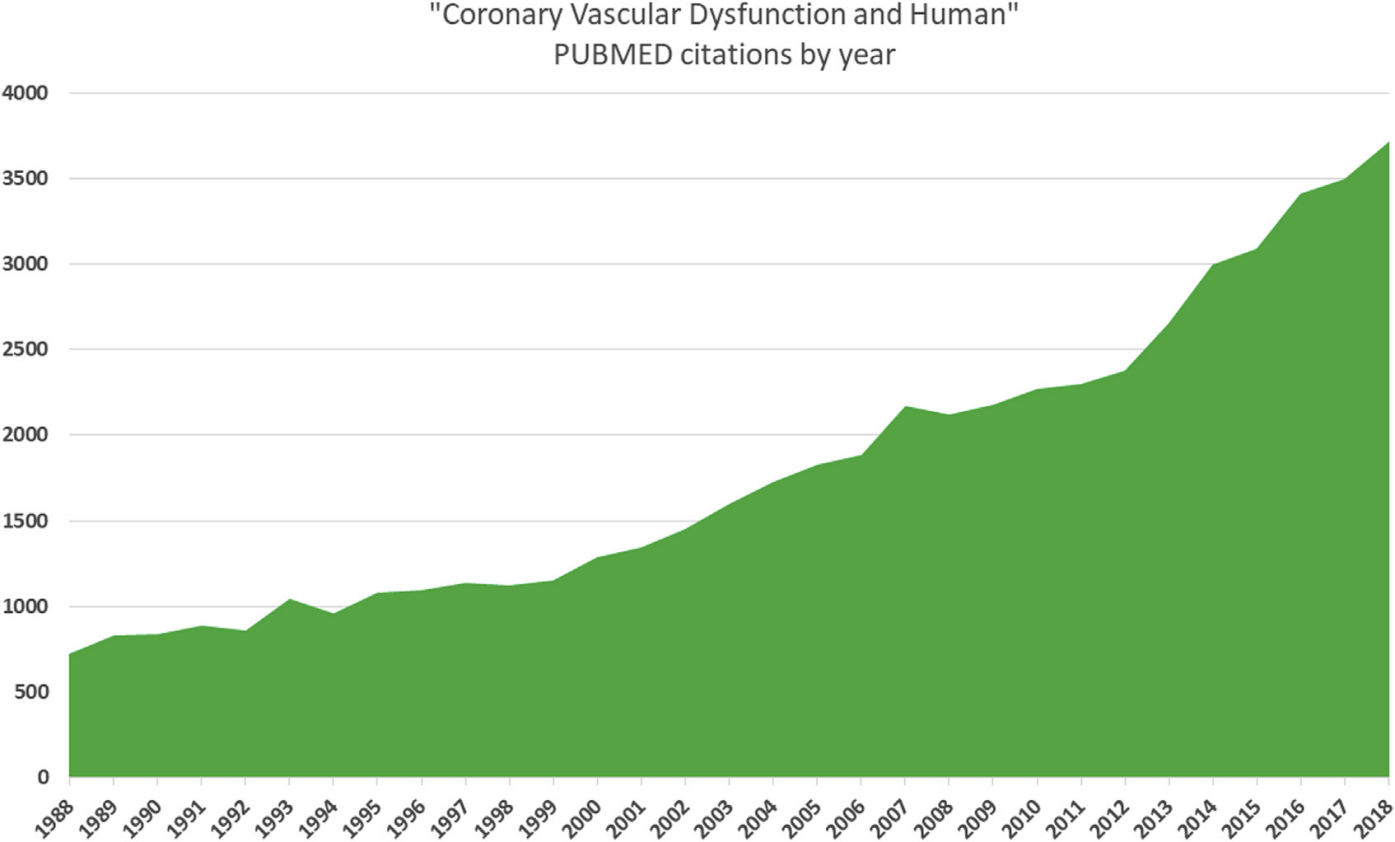
Rising Trend in Citations in Human Coronary Vascular Physiology A stacked area chart depicting the magnitude of change in citations between 1988 and 2018 and total values across this time period. Citations of “coronary vascular dysfunction and human” (https://www.ncbi.nlm.nih.gov/pubmed/?term=coronary+vascular+dysfunction+human; search date February 2, 2020).

There is a growing body of research underpinning the rationale for clinical tests of coronary vascular function (Figure 3). The clinical indication during coronary angiography should be personalized and considered on a case-by-case basis. A benefit/risk ratio applies. Benefits to patients, health care providers, and insurers relate to making the correct diagnosis with linked therapy (personalized medicine) and avoidance of inappropriate treatments and/or downstream investigations. An adjunctive IDP carries theoretical risks and prolongs the duration of the procedure, usually by 10 to 30 min. Staff training and experience can help optimize patient flow through the catheterization laboratory.

### Indications

The suggested clinical indications for an IDP are listed in Table 3. The indications align with the classification of MVA by Camici and Crea (96). In patients with stable symptoms, CorMicA provides proof-of-concept evidence that stratified therapy guided by results from an adjunctive IDP may be beneficial to patients with INOCA. Larger scale studies will be needed to substantiate new practice guideline recommendations. Pharmacological and nonpharmacological measures together bring patient benefits (97).

**Table 3:** Indications for Measuring Coronary Vascular Function as an Adjunct to Clinically Indicated Coronary Angiography

Condition	Invasive Diagnostic Management	Abbreviation
Current indications		
Angina	No obstructive coronary disease	INOCA
Myocardial infarction	Infarction without culprit stenosis for which vasospastic angina is considered	MINOCA
Cardiac arrest	In certain scenarios (ventricular arrhythmias, resuscitated cardiac arrest) for which no clear cardiac cause can be found and the patient is stabilized with normal LV function, no obstructive CAD, and normal ECG findings	VSA
Future possibilities		
Angina	Suspected obstructive CAD	Pre-PCI
	Post-PCI	Post-PCI
Heart failure	Preserved systolic function	HFpEF
	After cardiac transplantation	
Myocardial infarction	Stratification of risk and prognosis	STEMI, NSTEMI
	No obstructive coronary disease	MINOCA

CAD = coronary artery disease; ECG = electrocardiographic; HFpEF = heart failure with preserved ejection fraction; INOCA = ischemia with no obstructive coronary artery disease; LV = left ventricular; MINOCA = myocardial infarction with no obstructive coronary disease; NSTEMI = non–ST-segment elevation myocardial infarction; PCI = percutaneous coronary intervention; STEMI = ST-segment elevation myocardial infarction; VSA = vasospastic angina.

Coronary vascular dysfunction is implicated in the pathogenesis of several forms of cardiac disease, notably stable ischemic heart disease, acute MI, hypertension, diabetes, nonischemic cardiomyopathies, and heart failure with preserved ejection fraction. In patients with acute ST-segment elevation MI, multiple studies provide evidence supporting the prognostic value of IMR and CFR when measured at the end of percutaneous coronary intervention, and clinical trials of stratified medicine on the basis of IMR are ongoing (98). There is some evidence that coronary vascular dysfunction is implicated in the pathophysiology of MINOCA (99). More research on how vascular function testing may associate with treatment response within endotypes of INOCA and MINOCA is needed. Recent evidence supports a plausible role for targeted therapy (endothelin receptor A antagonists) modulating the endothelin-1 system in coronary microvascular dysfunction (100).

In heart failure with preserved ejection fraction, coronary microvascular dysfunction is implicated (69), notably in patients with cardiovascular risk factors such as hypertension. Presently, there are no evidence-based treatments for heart failure with preserved ejection fraction, but coronary microvascular dysfunction could become a treatment target. Coronary microvascular dysfunction is also implicated in cardiac transplant vasculopathy, and angiotensin-converting enzyme inhibitors may be beneficial (101).

## Conclusions and Future Directions

Contemporary practice guidelines state that in patients with anginal symptoms and no obstructive coronary arteries, guidewire-based CFR and/or microcirculatory resistance measurements should be considered (Class IIa), and pharmacological tests may be considered (Class IIb) (9). In this review, we described the available techniques, practical considerations, and relevant clinical scenarios in which an IDP may be useful. Adopting an IDP empowers cardiologists allowing personalized medicine for individual patients. Stratifying undifferentiated patients in the clinic will pave the way for new insights into vascular mechanisms and disease-modifying therapy.

Diagnostic advances are emerging, notably measurement of absolute myocardial resistance (102). The Achilles’ heel of anatomic imaging with CTCA is the lack of information on vasomotor function. The extent and clinical significance of false-negative results in patients with angina and no obstructive CAD is currently being investigated (CorCTCA [Coronary Microvascular Function and CT Coronary Angiography]; NCT03477890) (103). Technological advances are needed, and noninvasive coronary microvascular disease strategies (e.g., positron emission tomography, cardiovascular magnetic resonance) are emerging. Developing evidence-based, disease-modifying therapy is a priority (54). To that end, randomized controlled trials of novel and repurposed drugs, as well as precision medicine, hold future promise.
